# Capturing Subtle Neurocognitive Differences in Children with and without Tourette Syndrome through a Fine-Grained Analysis of Design Fluency Profiles

**DOI:** 10.3390/jcm11071946

**Published:** 2022-03-31

**Authors:** Mélina Tessier, Annie Desmarais, Julie B. Leclerc, Marc E. Lavoie, Kieron P. O’Connor, Bruno Gauthier

**Affiliations:** 1 Département de Psychologie, Université de Montréal, Montréal, QC H2V 2S9, Canada; melina.tessier@umontreal.ca (M.T.); annie.desmarais.3@umontreal.ca (A.D.); 2 Centre de Recherche de l’Institut Universitaire en Santé Mentale de Montréal, Montréal, QC H1N 3V2, Canada; leclerc.julie@uqam.ca (J.B.L.); marc.lavoie@umontreal.ca (M.E.L.); 3 Département de Psychologie, Université du Québec à Montréal, Montréal, QC H2X 3P2, Canada; 4 Département de Psychiatrie et d’Addictologie, Université de Montréal, Montréal, QC H3T 1J4, Canada; 5 Laboratoire de Psychophysiologie Cognitive et Sociale, Centre de Recherche de l’Institut Universitaire en Santé Mentale de Montréal, Montréal, QC H1N 3V2, Canada

**Keywords:** Tourette syndrome, executive function, design fluency, Five-Point Test, ADHD, clinical neuropsychology, child

## Abstract

Background: Tourette syndrome (TS) can be accompanied by neurocognitive impairment. Only a few studies have focused on executive function assessment in TS using design fluency, providing preliminary results. This study aimed to characterize the detailed design fluency profile of children with TS compared with neurotypical children, while addressing the central concern of frequent comorbidities in studies on TS by considering tic severity and attention-deficit/hyperactivity disorder (ADHD) symptoms and diagnosis. Methods: Sixty-one children aged between 6 and 15 years participated and were divided into a TS group (*n* = 28 (with ADHD *n* = 15)) and a control group (*n* = 33). Our objective was addressed by examining a wide range of measures of the Five-Point-Test, presumably sensitive to frontostriatal dysfunction. The total number of designs, repetitions, repetition ratio, unique designs, and numerical, spatial, and total strategies were examined for the total duration of the test (global measures) and at five equal time intervals (process measures). Results: The TS group produced significantly fewer numerical strategies. Groups did not differ in other global or process measures. ADHD did not affect performance. Conclusions: Children with TS do not inherently show general executive dysfunction but may present with subtle neurocognitive characteristics here revealed by comprehensive design fluency profiles.

## 1. Introduction

Children with Tourette syndrome (TS) are characterized by multiple motor tics and at least one phonic tic, which must be present for at least one year and expressed as simple or complex tics. Simple tics are defined as repetitive non-voluntary contractions of functionally related groups of skeletal muscles in one or more parts of the body, including blinking, cheek twitches, head or knee jerks, and shoulder shrugs [1]. Complex tics may take the form of a series of movements involving several muscle groups such as facial grimacing, tense–release hand gripping cycles, finger twiddling, or self-inflicted repetitive actions such as head-slapping, face scratching, tense–release hand gripping cycles, or finger twiddling. Evidence suggests that TS may result from an under-activation of basal ganglia structures at the neurofunctional level. This underactivation results in circuit dysfunctions that connect this subcortical structure to the frontal cortex, the so-called cortical-striatal-thalamocortical (CSTC) loop [2,3,4,5,6]. These CSTC circuit anomalies could also impact some neuropsychological functions in TS individuals [7,8,9]. Comorbidity is associated with TS in approximately 90% of patients [10,11,12], attention-deficit/hyperactivity disorder (ADHD) being one of the most common [13]. Despite high comorbidity rates, previous findings suggest a specific cognitive profile in children with TS, showing mild attention, visuomotor integration, fine motor, and executive function (EF) deficits [6,9,14]. Since EF involves prefrontal and subcortical structures, it seems reasonable to expect that the structural and functional abnormalities of the CSTC loop found in TS might explain, at least in part, the tic expression and lead to EF deficits [15,16].

Recent evidence suggests that children with TS may have specific impairments within particular EF sub-domains [7,17], the most frequently reported being inhibition, cognitive flexibility, planning, and verbal fluency deficits [9]. EF also includes design fluency (DF), the ability to generate diverse new ideas and solutions in the visuospatial domain [18]. Jones-Gotman and Milner [19] developed the first DF test analogous to verbal fluency. The task involves finding strategies to maximize the production of designs, while avoiding repeating patterns [20,21,22,23]. Successful performance, therefore, requires good visuomotor coordination as well as coordination of several EF, such as planning, initiation and self-monitoring skills, cognitive flexibility, inhibition of inappropriate responses, use of strategies, and creativity [24,25,26,27].

The most frequently used measure of DF performance is the total number of drawings. However, this measure alone is not sufficient to appreciate all aspects involved in such a multifactorial task [22]. It is possible to explore the underlying processes involved in DF more directly by considering the number of repetitions and unique designs [20,23]. A ratio of repetitions can also be computed by dividing the number of repetitions by the total number of drawings, a more sensitive measure than the number of repetitions, to identify patients with frontal lobe dysfunctions [28,29]. Another interesting measure concerns the strategies used to complete the task, of which there are two types: spatial (design rotation and mirroring) and numerical (line addition and subtraction) [30]. Finally, all those variables can be observed at different intervals (e.g., every minute) to look at the dynamic aspect of performance and compare these process measures to overall performance. The former may be more sensitive to detecting a DF anomaly, such as observed in ADHD [31].

Previous studies have highlighted interesting characteristics of DF. Research suggests that DF performance is sex-independent [28,32,33,34]. Furthermore, DF performance improves linearly with age [33,35,36,37,38,39]. In adults, DF is sensitive to frontal lobe functioning and frontostriatal lesions [29,40,41,42,43]. Knowing that TS is likely accompanied by frontostriatal dysfunction, DF could help document specific executive functioning deficits in this population.

In addition to gaining a better understanding of cognitive functioning, the study of DF in a TS population allows the sort of deficits often observed in neurological conditions to be investigated, including frontal lobe dysfunction and other similarities with TS such as ADHD and obsessive-compulsive disorder (OCD) [21,31,44,45,46,47]. To date, only two studies have examined DF in TS. In the first one, no significant difference was detected in terms of errors and strategies between TS, ADHD, and neurotypical children (*n* = 74 between the ages of 6 and 16 years), but these results were attributed to low statistical power [48]. The second study failed to find any DF deficit (i.e., unique designs and repetitions) in adults (*n* = 14 between the ages of 18 and 62) with TS/OCD comorbidity [49]. These findings remain preliminary as they involve small samples and a limited number of DF measures, thus requiring further replication.

The current study aims to characterize the design fluency profile of TS children with and without ADHD compared with neurotypical children (i.e., without TS). This characterization will be addressed by integrating a wide range of design fluency measures and analyzing overall performance (i.e., global measures) and temporal performance (i.e., process measures). Since comorbid disorders in TS represent a major confounding factor in neuropsychological studies [9], and given that the neurological anomalies observed in TS correlate with tic severity [50], we may expect such factors to be associated with cognitive impairments beyond the mere presence of a tic disorder. Furthermore, given the paucity of studies on design fluency in TS, the detailed analysis of the present study will contribute to the literature on the subject, add to existing research on the DF profiles in clinical pediatric populations (see [21,31,46,51,52,53]), and thus might provide evidence to guide clinicians through assessment.

## 2. Method

### 2.1. Participants

Sixty-one children aged between 6 and 15 years old (M = 10.3) were recruited and divided into a group of 28 children (19 boys) with TS and a group of 33 neurotypical children (19 boys). To be eligible, children with TS needed to be free of serious social, behavioural, and family problems, in addition to having a simple or complex daily tic for at least a year. The exclusion criteria were: major medical conditions, head injury, autism spectrum disorder, intelligence quotient (IQ) lower than 75, other neurological or psychiatric disorders (mild comorbidity of anxiety and depression not requiring treatment were not excluded), drug or alcohol abuse, active psychotherapy, acupuncture, hypnotherapy, and massage therapy. Psychiatric disorders were assessed using the Behavioral Assessment Scale for Children 2nd Edition [BASC-2]) [54]. In addition, participants who were stabilized on medication at the time of the study were included (stabilization on medication implied receiving typical or atypical neuroleptic medication or antidepressant or psychostimulant medication over at least three months with no further improvement in symptoms and a willingness to keep dosage constant throughout the study). The TS group included children with (*n* = 15) and without ADHD (*n* = 13). For the control group, the exclusion criteria were focused on head injury, neurological disorders, and DSM-5 diagnoses [1], including neurodevelopmental, emotional, behavioural, and conduct disorders.

### 2.2. Procedure

This study forms parts of a larger randomized controlled clinical trial that compares two types of cognitive-behavioural therapies in children and adults with TS (NCT03225430; https://clinicaltrials.gov/ct2/show/NCT03225430, accessed on 31 January 2022). The current study was coordinated at the *Centre d’Études sur les Troubles Obsessionnels-Compulsifs et les Tics* (CÉTOCT) based at the Research Center of the *Institut Universitaire en Santé Mentale de Montréal* (IUSMM). An initial phone interview with the parent assessed the child’s eligibility based on geographical accessibility and the absence of exclusion criteria. Inclusion criteria were then assessed with a structured clinical interview where a neurologist assessed the tics and severity of TS. Next, parents signed a consent form and completed TS and ADHD symptoms questionnaires. Finally, the child with TS was invited for a two-hour neuropsychological assessment that began with administering the design fluency test, then by an estimate of verbal and non-verbal IQ and other EF, attention, and fine motor skills tests. Although the control and clinical groups did not come from the same research project, the procedure was the same for the control group. The child was met for a two-hour assessment, including the DF test, verbal and non-verbal IQ estimate, and other EF tests.

### 2.3. Assessment Instruments

#### 2.3.1. Five-Point Test

**Figure 1:**
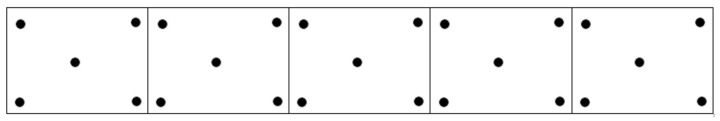
Example of Stimuli from the Five-Point Test.

This paper–pencil test is a simple and easy-to-understand tool for assessing DF [30] with strong psychometric properties (construct validity, reliability, internal consistency) and the administration and correction protocol of which are standardized [18,55,56]. It includes an 8.5 × 11-inch sheet with 40 rectangles arranged as eight rows of five squares, each containing five dots placed as on the surface of a dice (Figure 1).

The child was instructed to draw in each rectangle by connecting dots. Three specific rules were given: (1) make a different design in each rectangle; (2) connect points with straight lines; and (3) each line starts and ends on a point. The evaluator presented two examples of acceptable designs and mentioned that the test lasts five minutes and that it is not necessary to use all points in a rectangle. A second sheet was provided if required. Each measure was scored at each minute to analyze the temporal performance based on these five-time intervals and global scores. Thus, for each interval and the total test duration, the variables analyzed are: (a) (total designs) to measure productivity and initiation; (b) (repetitions) to measure self-regulation, i.e., cognitive flexibility and inhibition (drawing was considered a repetition if it has already been performed in the same or a previous interval); (c) (repetition ratio) to assess repetitions taking into account productivity; (d) (unique designs), i.e., total minus repeated designs, a measure that reflects both productivity and self-regulation; (e) (strategies), combining the number of (spatial strategies: rotation and mirror operations on the previous design) and (numerical strategies: adding and subtracting a line from the previous design), reflecting planning. Strategies that involved repetitions were counted.

#### 2.3.2. IQ Estimate

Standardized scores (M-10, S.D.-3) of the Wechsler children’s intelligence scale Vocabulary and Matrix reasoning subtests—5th edition: French-Canadian version (WISC-V CDN-F) [57] were used to estimate the IQ of children with TS, while standardized scores from the WISC-V CDN-F Similarities and Matrix reasoning subtests were used for the control group. These subtests are recognized as reliable measures of verbal and non-verbal reasoning. For instance, Vocabulary assesses the ability to define words presented orally; Similarities assesses the ability to identify a common abstract concept between two words; Matrix reasoning assesses the ability to identify patterns that logically complete graphic matrices. As already mentioned, the control group and the clinical group were not from the same study. WISC-V Similarities was used for the control group as it best correlates with full scale IQ (FSIQ). The TS group took part in a clinical trial in which the adult version of Vocabulary was administered to adult TS participants. WISC-V Vocabulary was therefore selected for continuity and possibly for developmental analyses. Nevertheless, both tests show very similar relationships with FSIQ and verbal IQ (VIQ), the intercorrelations corrected coefficients of Similarities with VIQ ranging from 0.57–0.77 and with FSIQ from 0.59–0.76, and of Vocabulary with VIQ ranging from 0.57–0.77 and with FSIQ from 0.51–0.74 for the ages of 6 to 15 years old [58].

#### 2.3.3. TS Symptoms

The Yale Global Tic Severity Scale (YGTSS) [59] was used to assess tic symptoms during the week previous to assessment. It identifies motor and vocal tics and evaluates the amount, frequency, intensity, and complexity of tics. Each dimension is assessed on a 0–5 scale, and three total scores are calculated: total motor tics (0–25), total vocal tics (0–25), and total severity score (0–50). The raw scores of the three total scores were used in the analyses.

#### 2.3.4. ADHD-Related Symptoms

ADHD-related symptomatology was assessed for children with TS using the Revised Conners for parents scale: short version (CPRS-R:S) [60]. This questionnaire for parents of 6- to 18-year-olds is based on the ADHD manifestations as described in the DSM-5 [1] and focuses on the child’s behaviour during the last month on a 0–3 scale (0—Never/rarely; 1—occasionally; 2—Often/Often Enough; 3—Very often). T-scores (M-50, S.D.-10) for scale B (inattention), scale C (hyperactivity) and scale D (DA-H inattention-hyperactivity deficit) were used in the analyses.

### 2.4. Statistical Analyses

All analyses were made with IBM SPSS (version 25). Considering the study’s exploratory nature, interpretations were performed with the conventional significance level of 0.05 without correcting for multiple comparisons or correlations. To describe the two groups and identify confounding variables, we conducted independent-group *t*-tests to compare age and IQ (verbal and non-verbal) and a Chi-square test to verify sex equivalence. Pearson correlations between demographic and FPT variables were computed (combined for all participants and separately for each group). Then, to compare the FPT performance of both groups, a descriptive analysis of the FPT variables was carried out. Independent sample *t*-tests and analyses of variance (ANOVA) were performed, and Pearson correlations were used to analyze the degree to which the various FPT variables are related. Finally, to compare FPT performance time course across groups over the five minutes (i.e., five intervals), a repeated-measures analysis of variance (ANOVA) was performed separately for each FPT variable. Missing data were replaced by the mean. We conducted all inferential analyses with two groups (TS and Control) and three groups (TS with ADHD, TS without ADHD, and Control). The results were the same, and the two-group results are reported here.

## 3. Results

### 3.1. Group Equivalence and Covariates Identification

The two groups did not differ significantly on non-verbal IQ, verbal IQ, and age (Table 1). The Chi-square test showed no significant difference between groups in terms of sex. Pearson correlations revealed that the TS group’s non-verbal IQ significantly correlated, with small-size effects [61], with total designs (r = 0.44, *p* = 0.020) and unique designs (r = 0.44, *p* = 0.019), while the control group non-verbal IQ did not correlate with any FPT variable. When participants were grouped together, non-verbal IQ correlated significantly with total designs (r = 0.30, *p* = 0.017), unique designs (r = 0.34, *p* = 0.007), spatial strategies (r = 0.27, *p* = 0.033), and total strategies (r = 0.28, *p* = 0.029). Age correlated significantly (average effect size) with all FPT variables, apart from numerical strategies, repetitions, and its ratio (non-significant, small-size effect) (Table 2).

### 3.2. FPT Performance

The mean performances of each group in terms of global measures on the FPT are reported in Table 3. Results of analyses of variance (ANOVA) showed significant intergroup difference for numerical strategies (F(1, 59) = 6.56, *p* = 0.013, partial η^2^ = 0.100), but failed to show significant intergroup difference for total designs (F(1, 59) = 2.33, *p* = 0.132, partial η^2^ = 0.038), repetitions (F(1, 59) = 1.01, *p* = 0.319, partial η^2^ = 0.017), ratio of repetitions (F(1, 59) = 0.037, *p* = 0.848, partial η^2^ = 0.001), unique designs (F(1, 59) = 1.78, *p* = 0.188, partial η^2^ = 0.029), spatial strategies (F(1, 59) = 0.079, *p* = 0.780, partial η^2^ = 0.001), and total strategies (F(1, 59) = 1.06, *p* = 0.308, partial η^2^ = 0.018).

### 3.3. FPT Process Measures

**Figure 2:**
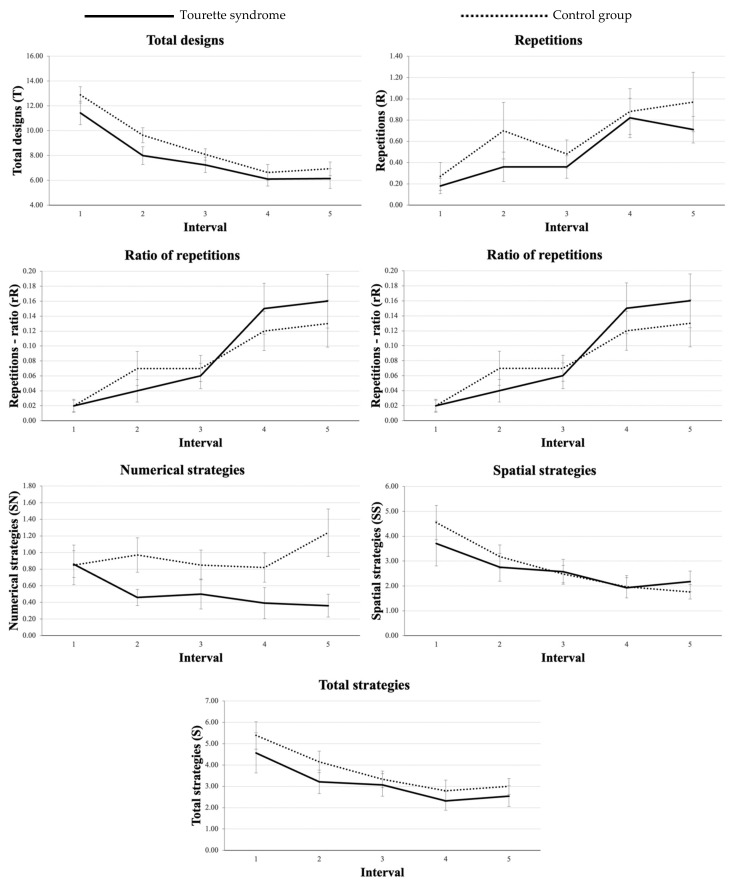
Averages of DF scores in function of intervals for Total designs, Unique designs, Repetitions, Repetition ratio, Numerical strategies, Spatial strategies, and Total strategies for each one-minute FPT interval. Error bars represent the standard error of the mean.

For all variables, results showed no significant interaction between groups and intervals, suggesting that the evolution of performance over time did not differ between groups. Results are presented in Table 4, and group-specific production patterns are shown in Figure 2.

### 3.4. A Posteriori Analyses

Given the discrepancies between verbal and non-verbal IQ in the TS group, *t*-tests for matched samples were carried out. For the TS group, there was a significant difference between verbal and non-verbal IQ, to the detriment of the latter (t(27) = 3.15, *p* = 0.004), while no significant difference was observed for the control group (t(32) = 1.34, *p* = 0.189).

## 4. Discussion

The purpose of this study was to document the detailed design fluency profile of children with TS with or without ADHD. Given the previously documented executive deficits in TS [9,15], we predicted a reduced performance on the Five-Point-Test of these children compared to neurotypical children. Overall, our results do not confirm this hypothesis, as we observed no difference between the TS and Control group regarding all process measures and all but one global measure. During the five-minute FPT, children with TS with or without ADHD produced significantly fewer numerical strategies than the control group. This performance partially corroborates the study of Mahone and colleagues [48], who observed a trend towards intergroup differences in the number of strategies and errors, TS children offering lower scores than neurotypical children. However, it should be noted that this result, although statistically significant, seems less so from a clinical perspective since the TS group obtained a low average score for numerical strategies when comparing their mean to that of the control group.

### 4.1. Decreased Performance of the Clinical Group at the FPT

In order to understand the results obtained for the TS group on design fluency performance measures, let us return to Bornstein and colleagues [62]. They raised the question of the relationship between symptoms such as tics, agitation, academic and behavioural problems and the often reduced neuropsychological performance observed in children with TS. They proposed a first hypothesis, according to which, both symptoms and cognitive deficits reflect the underlying pathophysiology of TS. A second hypothesis suggests that the symptoms per se interfere with performance during the assessment and, therefore, this so-called cognitive deficit constitutes a side effect of these symptoms. In the current study, the group equivalence with regard to age, sex, and IQ, together with the lack of correlation between ADHD symptoms, the severity of tics, and FPT performances, suggest that the lower score of the TS group on numerical strategies is not due to tics but may instead reflect the frontostriatal dysfunction inherent to the disorder. In other words, the anomaly of the cortico-subcortical network could interfere with the optimization of complex cognitive performance [9,63] as required by the FPT, without any relation to symptom intensity.

Furthermore, our results support the multidimensional nature of design fluency and, therefore, the usefulness of considering several measures in addition to the overall score when interpreting an FPT performance. The present results support the relevance of separating numerical strategies from spatial strategies. The TS group did not produce significantly fewer spatial strategies than the control group, as is the case for numerical strategies. Those two strategy indices were introduced by Goebel and colleagues [64] and studied separately for the first time with children by Hurks and colleagues [65]. The dissociation observed in our results supports the relevance of considering those two measures separately when interpreting FPT performances with children. The present study supports the need for further investigation of the individual contribution of each type of strategy and considers a possible double dissociation to understand their relevance to the interpretation of FPT results.

The fewer numerical strategies suggest that children with TS might have reduced executive skills, including planning. The present results suggest that the number of numerical strategies could be a sensitive measure to reflect a subtle weakness at this level in children with TS. These results could also be related to attention regulation and vigilance deficits, which would explain the weaker ability to implement various strategies during a task requiring important attentional control, such as the FPT. Attentional dysregulation is an attribute of TS that is part of the cognitive-behavioural/psychophysiological model of tic disorders proposed by [66]. According to this model, inattention is due to the chronic brain overactivation associated with TS, in addition to being potentially exacerbated by perfectionist tendencies that often accompany the disorder, since future-oriented thoughts result in a reduced attentional focus on the task [66,67]. The model also stipulates that cognitive factors play a central role in the emergence and maintenance of tics and that the modification of these symptoms could be made through an intervention on two sources at the origin of tics; one cognitive/emotional and the other behavioural/physiological [68,69,70]. The impact of such therapy on cognition and design fluency should be addressed in future studies.

### 4.2. Intact Time Domain of Design Fluency Task in Tourette Patients

The added value of the current study also lies in its analysis of process measures as well as the overall performance. Performance evolution in time intervals is similar across groups, whereas the number of designs and strategies decreased over time while repetitions increased. The same production pattern has been observed in neurotypical adults [71]. Thus, our results indicate that children with TS differ from neurotypical children on a measure of overall FPT performance, as shown for the numerical strategies, but not on process measures. Performance becomes increasingly demanding for all children over time, a pattern previously observed in neurotypical children [31], but that has never been previously analyzed in this clinical group.

### 4.3. Verbal vs. Non-Verbal Discrepancy in Children with TS

Our results significantly differ between verbal and non-verbal IQ estimates uniquely in the TS group. These results are consistent with previous studies on intellectual functioning in TS that report an IQ between low average and average in this population [9]. The discrepancy between verbal and non-verbal IQ in TS patients is also documented, with a difference of 15 points or more observed in 25–55% of TS samples, compared with approximately 10% in neurotypical samples [62,72,73,74,75,76]. This discrepancy is often found in patients with bilateral or right hemisphere dysfunction, and the high occurrence of this discrepancy in TS has been interpreted as evidence of brain dysfunction [77,78]. Two more recent studies have assessed IQ in TS, one in children but only reporting full scale IQ [79] and the other observing no difference between verbal and performance IQ, but in adults [80]. Recent studies in the neuropsychology of Tourette syndrome are scarce and particularly those contrasting groups with nonverbal materials and conclusions must be interpreted with caution.

### 4.4. Developmental Aspects

The correlational analyses revealed a positive correlation between age and all FPT variables, except for repetitions and its ratio measure, and no relation with sex, confirming earlier studies showing an effect of age but no sex difference in design fluency performances [22]. The relationship between age and design fluency is consistent with studies on cognitive development in children and the FPT’s usefulness in characterizing this development [21,22,35,39]. Compared with inhibition (see [35,81,82] and cognitive flexibility [83], fluency would develop late as it actively involves strategizing, monitoring, and self-assessment. Therefore, it is considered a high-level, complex, and multimodal neuropsychological process, which first requires effective functioning of attention, inhibition, and other executive functions involved in performance [35]. The development of design fluency likely continues during adolescence and early adulthood [33,82,84]. Thus, between ages of 7 and 11, children first become more strategic, organized, and effective in their performance, while strategy refinement continue to evolve during adolescence [83].

### 4.5. Limitations

Only tics and ADHD symptoms and diagnosis were examined in the current study, while TS is frequently associated with other disorders that are likely to influence neuropsychological performance, such as OCD, anxiety, depression, and learning and behavioural disorders. Some studies suggest that the onset of tics may be associated with increased neuropsychological impairment [62,73,85] and that parental education is related to design fluency [35]. Thus, further studies are required to assess the influence of these variables on design fluency. The effect of drug therapy was also not examined and should be investigated, especially for medication known to affect executive development. Finally, multiple analyses increase the risk of obtaining a significance threshold by chance, another limitation of the study.

## 5. Conclusions

To our knowledge, this is the first study to investigate design fluency in children with TS, incorporating several global and process performance measures of the FPT. Our results suggest that the performance of children with TS does not differ in terms of process measures but that the detailed analysis of performance measures (numerical strategies most notably) might help reveal subtle cognitive fragility in TS. Although the FPT is a complex task involving many components and requiring the coordination of several cognitive functions, each FPT measure might specifically identify an executive deficit, such as planning, initiation, self-monitoring, or inhibition. From a clinical point of view, professionals need to be aware of specific cognitive weaknesses of children with TS to individualize and optimize intervention. However, it should be noted that no significant deficit has been revealed with the FPT.

In addition, our findings add to the growing evidence that TS is not associated with generalized deficits of executive functions but that relatively limited executive weaknesses might be present, regardless of the severity of tics and the presence of ADHD. Our results contribute to the existing literature on TS’s neurocognitive profile and support the existence of a significant discrepancy between verbal and non-verbal IQ. TS appears to involve a cluster of multiple cognitive characteristics, and further research on cognitive aspects of the disorder will undoubtedly lead to a better understanding of the different clinical phenotypes of patients with TS.

## Figures and Tables

**Table 1:** Descriptive and Clinical Data.

Variables	TS (*n* = 28)			Control (*n* = 33)			*p*
Male (%)	19 (67.9%)			19 (57.6%)			ns
	M (SD)	Min	Max	M (SD)	Min	Max	
Age (years, months)	10.7 (1.9)	6.7	13.11	10.1 (1.9)	7.4	15.3	ns
Verbal IQ	12.4 (3.0)	6	18	12.2 (2.5)	7	19	ns
Non verbal IQ	10.9 (2.5)	6	18	11.6 (2.3)	7	16	ns
Age of 1st tic (months)	5.9 (2.4)	1.0	9.7				
YGTSS—motor tics	11.1 (3.8)	0	17	-	-	-	-
YGTSS—phonic tics	6.6 (4.4)	0	16	-	-	-	-
YGTSS—total score	30.6 (15.2)	7	67	-	-	-	-
Conners—Inattention	61.5 (12.0)	42	90	-	-	-	-
Conners—Hyperactivity	62.9 (13.7)	44	90	-	-	-	-
Conners—AD-H	61.4 (14.7)	9	88	-	-	-	-

Note: TS = Tourette syndrome; AD-H = attention deficit-hyperactivity; M = mean; SD = standard deviation; Min = minimum; Max = maximum; ns = non-significant.

**Table 2:** Pearson Correlations between Demographics, including TS and ADHD symptoms, and FPT Global Measures.

					Strategies		
	Total Designs	Repetitions	Ratio Repetitions	Unique Designs	Numerical	Spatial	Total
Age	0.376 **	−0.083	−0.243	0.418 **	0.186	0.303 *	0.340 **
TS	0.479 **	0.004	−0.371	0.490 **	0.074	0.439 *	0.418 *
Controls	0.344 *	−0.101	−0.172	0.418 *	0.353 *	0.167	0.308
Sex	0.038	0.123	0.140	0.008	−0.159	0.073	0.021
TS	0.023	−0.121	0.047	0.038	−0.276	0.075	0.022
Controls	0.014	0.200	0.190	−0.069	−0.178	0.066	−0.009
Verbal IQ	0.050	−0.110	−0.152	0.082	0.051	0.077	0.088
TS	0.064	−0.115	−0.154	0.079	0.076	0.101	0.107
Controls	0.050	−0.121	−0.159	0.105	0.064	0.049	0.074
Non-verbal IQ	0.304 *	−0.075	−0.171	0.340 **	0.075	0.273 *	0.279 *
TS	0.437 *	0.047	−0.212	0.441 *	0.145	0.341	0.341
Controls	0.098	−0.162	−0.161	0.174	−0.025	0.191	0.176
Age of 1st tic	−0.248	0.262	0.321	−0.286	−0.246	−0.317	−0.338
YGTSS—motor tics	0.042	−0.359	−0.345	0.086	−0.156	0.204	0.159
YGTSS—phonic tics	−0.211	0.242	0.340	−0.245	0.151	−0.314	−0.263
YGTSS–total	0.006	0.029	0.002	0.002	0.169	0.009	0.041
Conners—Inattention	−0.002	−0.023	−0.011	0.000	−0.006	−0.035	−0.029
Conners—Hyperactivity	0.320	0.127	−0.076	0.312	0.132	0.252	0.255
Conners—AD-H	0.127	0.179	0.139	0.109	0.109	0.011	0.033

Note. TS = Tourette syndrome; AD-H = attention deficit-hyperactivity; * *p* < 0.05; ** *p* < 0.01.

**Table 3:** Mean Performance of TS and Control Groups for each Global Measures on the FPT.

	M (SD)		Min		Max	
Global Measures	TS	Controls	TS	Controls	TS	Controls
Total designs (T)	38.93 (15.63)	44.18 (11.18)	12	19	80	65
Repetitions (R)	2.43 (1.83)	3.30 (4.28)	0	0	7	24
Repetitions-ratio (rR)	0.07 (0.05)	0.07 (0.07)	0	0	0.17	0.37
Unique designs (U)	36.50 (15.28)	40.88 (10.23)	10	18	79	60
Numerical strategies (NS)	2.57 (2.30)	4.73 (3.92)	0	0	8	18
Spatial strategies (SS)	13.18 (11.89)	13.94 (9.25)	0	0	52	36
Total strategies (S)	15.71 (12.89)	18.67 (9.51)	1	3	55	38

**Table 4:** Results of the Repeated-Measures Analysis of Variances.

	I1	I2	I3	I4	I5	Interval		Group		Interaction (Interval × Group)	
	M (SD)	M (SD)	M (SD)	M (SD)	M (SD)	*p*	η^2^_partial_	*p*	η^2^_partial_	*p*	η^2^_partial_
Total designs (T)	12.2 (4.40)	8.89 (3.68)	7.70 (2.92)	6.39 (3.37)	6.57 (3.67)	<0.001 **	0.435	0.132	0.038	0.773	0.008
Repetitions (R)	0.230 (0.616)	0.541 (1.232)	0.426 (0.670)	0.852 (1.123)	0.852 (1.262)	<0.001 **	0.093	0.319	0.017	0.884	0.005
Repetitions-ratio (rR)	0.019 (0.044)	0.057 (0.108)	0.061 (0.094)	0.134 (0.168)	0.143 (0.183)	<0.001 **	0.167	0.880	0.000	0.592	0.012
Unique designs (U)	11.983 (4.319)	8.344 (3.568)	7.279 (2.972)	5.541 (3.243)	5.721 (3.503)	<0.001 **	0.480	0.188	0.029	0.840	0.006
Numerical strategies (NS)	0.853 (1.152)	0.738 (0.964)	0.689 (1.009)	0.623 (1.019)	0.836 (1.368)	0.651	0.010	0.013 *	0.100	0.164	0.027
Spatial strategies (SS)	4.164 (4.325)	2.984 (2.790)	2.525 (2.307)	1.951 (2.383)	1.951 (1.919)	<0.001 **	0.147	0.770	0.001	0.570	0.012
Total strategies (S)	5.016 (4.319)	3.721 (2.922)	3.213 (2.497)	2.574 (2.642)	2.787 (2.325)	<0.001 **	0.154	0.308	0.018	0.924	0.004

Note. I1, I2, … = Interval 1, interval 2, …; M = mean; SD = standard deviation; η^2^_partial_ = partial eta-square; * *p* < 0.05, ** *p* < 0.01.
